# Repurposing of FDA‐Approved Drugs to Disrupt Iron Uptake in *Mycobacterium abscessus*: Targeting Salicylate Synthase as a Novel Approach

**DOI:** 10.1111/cbdd.70162

**Published:** 2025-07-29

**Authors:** Giovanni Stelitano, Christian Bettoni, Matteo Mori, Mario Cocorullo, Andrea Tresoldi, Fiorella Meneghetti, Stefania Villa, Laurent R. Chiarelli

**Affiliations:** ^1^ Department of Biology and Biotechnology “Lazzaro Spallanzani” University of Pavia Pavia Italy; ^2^ Department of Pharmaceutical Sciences University of Milan Milano Italy

**Keywords:** drug repurposing/repositioning, drug resistance, iron uptake inhibitors, nontuberculous mycobacteria, salicylate synthase, siderophores

## Abstract

Non‐tuberculous mycobacteria (NTM) are opportunistic pathogens that lead to severe, persistent infections, particularly in immunocompromised or vulnerable individuals. Infection rates are rising worldwide, highlighting NTM as an increasing threat to public health. There are currently no specific drugs, and the recommended regimens are usually ineffective. This scenario underlines the urgent need to develop new strategies to effectively combat these infections in a more innovative way. However, the development of new drugs can be a lengthy process, often taking more than a decade to identify even a single active compound. Among the new strategies that can expedite this process is the repurposing of approved drugs. In this work, we applied this approach to identify compounds inhibiting iron uptake in *Mycobacterium abscessus* (*Mab*). Specifically, we studied the targeting of salicylate synthase, an enzyme that plays a crucial role in the biosynthesis of mycobacterial siderophores necessary for iron acquisition. Performing an *in silico* virtual screening of three databases against the crystal structure of salicylate synthase, we identified 11 potential ligands. Then, in vitro assays on the recombinant enzyme highlighted three competitive inhibitors, namely fostamatinib, esomeprazole, and hydroxystilbamidine. These results confirm the potential of the repurposing approach and pave the way for further experimental validation and optimization of these inhibitors as promising compounds against NTM infections.

## Graphical abstract

A drug repurposing strategy was applied against *M. abscessus,* through a virtual screening of FDA approved drugs on salicylate synthase. Eleven potential ligands were found; among these, three were confirmed as potent inhibitors of the enzyme.

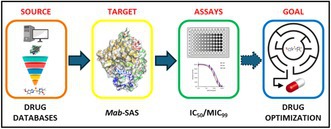

## 1 Introduction

Antimicrobial resistance poses a significant threat to public health and the global economy, undermining the effectiveness of existing treatments and complicating efforts to control infectious diseases. Bacteria may become resistant to antibiotics through a variety of intrinsic and acquired mechanisms, including epigenetic modifications, compartmentalization, changes in cell wall permeability, efflux pumps, and target mutations (Verma et al. 2024).

Over the past few decades, *Mycobacterium abscessus* (*Mab*), a member of the non‐tuberculous mycobacteria (NTM) group, has emerged as an opportunistic pathogen causing pulmonary infections, particularly in people with cystic fibrosis (CF) and chronic obstructive pulmonary disease (COPD) (Degiacomi et al. 2019); (Dedrick et al. 2023). *Mab* exhibits high resistance to current antimicrobial agents, making the available therapies, which are mostly based on old drugs, largely ineffective (Fröberg et al. 2023).

This scenario emphasizes the need to develop new drugs and strategies to fight bacterial infections more efficiently and in an innovative way. However, drug development is a lengthy process, often taking more than a decade to identify even a single active compound (Hardman et al. 2023). Nevertheless, new resources and strategies, such as molecular modeling tools and the repurposing of approved drugs, can accelerate this procedure (Schuler et al. 2022); (Savva et al. 2024); (Waseem et al. 2024). Drug repurposing refers to the process of identifying new therapeutic uses for existing drugs (typically FDA‐approved), with established information on their toxicity, formulation, pharmacology, and potential side effects. This approach offers a cost‐effective and time‐efficient alternative to traditional drug discovery. There are two main strategies for drug repurposing. The first involves discovering new uses for drugs by chance, a process known as “serendipity”. A classic example is botulinum toxin A (BoNT‐A), also known as Onabotulinumtoxin A, which was initially used for the treatment of strabismus and blepharospasm but is now approved for eight different medical applications (Israr et al. 2024). The second, more systematic approach focuses on the pharmaceutical, biological, and chemical properties of both the drug and its target. This involves analyzing the molecular characteristics of pharmacological targets to identify new potential applications for existing drugs, enabling a more direct and rational repurposing process. An example is azidothymidine (zidovudine), originally identified as a chemotherapeutic agent and then repurposed to fight the human immunodeficiency virus (HIV) (Tirelli et al. 1992). This approach often uses molecular modeling methods, such as docking and molecular dynamics, to explore the chemical/physical properties of the target. By applying quantum‐mechanical equations, this strategy helps identify the best ligands from a pool of approved drugs. Once promising candidates are selected, they are tested in vitro to confirm preliminary results. If successful, this approach can streamline the drug development process by potentially skipping or shortening some of the more costly phases, allowing researchers to move directly to animal models and Phase II clinical trials with a reduced risk of failure (Kulkarni et al. 2023).

This work aims to conduct drug repurposing studies to identify effective inhibitors of mycobactin biosynthesis in *Mab*, leading to the impairment of iron uptake. Iron is an essential cofactor for many *Mab* enzymes and virulence factors. For instance, iron is essential for the cytochromes involved in electron transport as well as hemoproteins involved in oxygen metabolism (De Voss et al. 1999), thus playing an important role during infection, replication, and biofilm formation (Mori et al. 2023). However, free iron is absent in the replicative niche of *Mab*, namely the alveolar macrophages. For this reason, the mycobacterium needs to scavenge the host's iron through specific siderophores, known as mycobactins. Belonging to the same genus as *M. tuberculosis* (*Mtb*), *Mab* shares many similar drug targets. Therefore, there is a great interest in the application of effective antituberculosis agents to *Mab* drug discovery, with the potential to identify anti‐infective agents that could be effective across various mycobacterial species. Given our research group's recent focus on developing *Mtb* drugs targeting siderophore biosynthesis (Mori et al. 2020); (Mori et al. 2021); (Mori et al. 2022), there is significant potential to extend our previous discoveries to *Mab*. In this work, we focused on inhibiting salicylate synthase (*Mab*‐SaS) through the repurposing of FDA‐approved drugs. The magnesium‐dependent *Mab*‐SaS, encoded by the gene *MAB_2245*, catalyzes the first reaction of the biosynthetic pathway of mycobactins, representing a particularly attractive drug target due to its absence in humans (Mori et al. 2023). Moreover, since this enzyme is a virulence factor, its inhibition does not create selective pressure as targeting essential genes would, thus helping to prevent the emergence of resistant mutants (Stelitano et al. 2023). Herein, we exploited computational tools to screen and select known drugs potentially inhibiting *Mab*‐SaS, with the goal to hinder iron uptake in *Mab* and circumvent antibiotic resistance.

## 2 Results and Discussion

Target‐to‐drug strategies have emerged as powerful approaches to accelerate the traditionally long and resource‐intensive process of drug development. We exploited this strategy by running an *in silico* analysis of FDA‐approved drugs to identify novel inhibitors of *Mab* salicylate synthase (*Mab*‐SaS), the first enzyme involved in mycobactin biosynthesis. We previously found that for this enzyme, the cofactor is not a key feature for the binding of the inhibitors (Mori et al. 2020). By focusing on FDA‐approved drugs, we aimed at bypassing many early‐phase development hurdles, such as safety profiling and toxicity testing, which are typically required for new chemical entities. The drug repurposing strategy takes advantage of the known pharmacokinetic and safety profiles of existing drugs, thus significantly reducing the time and cost associated with developing new antimicrobials.

Starting from the recent crystal structure of *Mab*‐SaS (Mori et al. 2024), a complete model was built and refined using Maestro. Missing amino acids were incorporated via homology modeling, and regions of the structure with low resolution, particularly flexible parts, were further optimized to improve their conformational accuracy. For the docking analysis, three databases of FDA‐approved drugs were sourced from Enamine, DrugBank, and PubChem. The molecules were prepared in their 3D conformations using LigPrep, which generated and optimized all possible stereoisomers, tautomers, and protonation states. This comprehensive approach ensured that all relevant conformers were considered, maximizing the likelihood of accurately capturing potential binding interactions during the docking process.

The docking results were filtered to remove duplicate entries. Selection of putative candidates was based on their docking positions (Figure S1), docking scores, and corresponding binding free energies. To further refine the selection, the MM‐GBSA (Molecular Mechanics Generalized Born Surface Area) binding free energies of the 14 top‐scoring molecules were calculated using the Prime module in Maestro. These results were used as an additional filtering criterion. Ultimately, 11 compounds were shortlisted for subsequent biological evaluation (Table 1).

**FIGURE 1:**
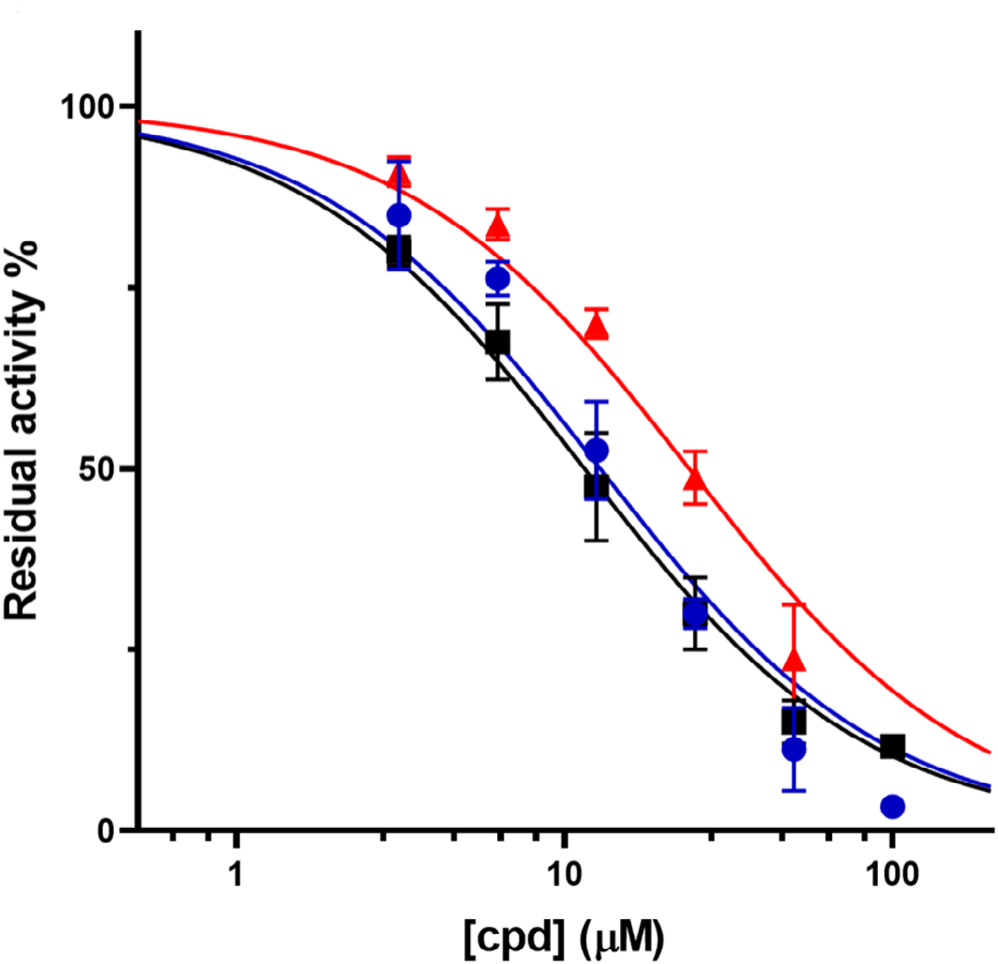
Inhibition assays of Mab‐SaS enzymatic activity by the three best candidates: IC_50_ determination for esomeprazole (blue circles), hydroxystilbamidine (black squares), and fostamatinib (red triangles).

**TABLE 1:** Compounds obtained by docking analyses performed against the model of *Mab*‐SaS. All compounds were docked into the active site, corresponding to the theoretical substrate‐binding position, and are ordered by their docking scores.

Compound	Structure	Docking score	MM‐GBSA (Kcal/mol)
CHO	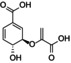	−7.75	−52.29
Nebivolol	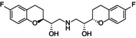	−8.1	−82.52
Hydroxystilbamidine	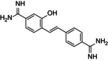	−8	−86.40
Cytidine	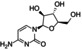	−7.92	−57.75
Gentamycin	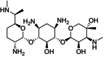	−7.6	−103.9
Norepinephrine	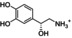	−7.4	−57.75
Fostamatinib	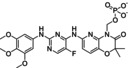	−7.4	−58.58
Labetalol	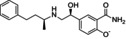	−7.29	−76.93
Levolansoprazole	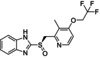	−7.7	−76.43
Esomeprazole	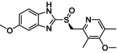	−7.2	−78.81
Amifostine	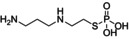	−6.9	−54.51
S‐adenosyl‐L‐homocysteine	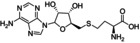	−6.8	−76.44

To confirm the relevance and reliability of the docking analysis findings, we evaluated the inhibitory activity of the selected molecules against Mab‐SaS. This experimental validation step was crucial to determine whether the predicted interactions from the computational analysis translated into measurable inhibition of the target enzyme. The activity assays were performed using a subsaturating concentration of chorismic acid (CHO; 70 μM), in the presence of 100 μM of each compound. Five out of 11 molecules showed a significant inhibitory effect against the enzyme (Table S1). At the tested concentration, esomeprazole, fostamatinib, and hydroxystilbamidine completely blocked the enzymatic activity, while labetalol and levolansoprazole showed a mild inhibition of about 53% and 37%, respectively. Finally, norepinephrine showed a modest inhibition of about 24%. It is noteworthy that all compounds exhibited distinct scaffolds, except for esomeprazole and levolansoprazole, which share a common core.

Additional assays were performed to ascertain that esomeprazole, hydroxystilbamidine, and fostamatinib specifically inhibit Mab‐SaS and do not behave as PAIN. Specifically, all the compounds inhibited the enzyme when BSA and Triton X‐100 were added, proving that they did not form aggregates with the target. Similarly, they did not interact with the cysteines, as the addition of DTT had no impact on the inhibition.

Speculating on why some molecules did not inhibit the enzyme, despite achieving favorable docking scores *in silico*, could provide valuable insights for future studies. For example, gentamycin was predicted to be a promising inhibitor of *Mab*‐SaS, with an estimated binding energy of −103.9 kcal/mol calculated using MM‐GBSA. This value is nearly twice as negative as the binding energy of CHO. Despite this promising predictive data, the compound did not exhibit any inhibitory effect in vitro, even at a concentration of 100 μM. This lack of inhibition may be attributed to the compound's excessive mobility, which could impede its ability to access the predicted binding site [18]. Similarly, the MM‐GBSA binding energy for S‐adenosyl‐L‐homocysteine was −76.44 kcal/mol, approximately 1.5 times more negative than that of CHO. Once again, this prediction did not reflect an inhibition effect on the enzymatic activity, possibly because its flexible core structure limits its ability to interact effectively with the enzyme.

Finally, the amifostine, which was predicted with a binding energy of −54.51 Kcal/mol, likely creates a circular structure through intramolecular interactions caused by the electronegativity of the phosphate moiety and the possible positive charges of amide groups, which may impair the entrance of the molecule into the active site and/or the binding with the target.

The three best candidates, namely esomeprazole, fostamatinib, and hydroxystilbamidine, were selected for further biological investigation against the target enzyme. The evaluation of the IC_50_ confirmed their good inhibitory properties against *Mab*‐SaS (Figure 1, Table 2).

**TABLE 2:** Evaluation of the best *Mab*‐SaS inhibitors identified by the computational procedure.

Compound	IC_50_ (μM)	K_i_ (μM)
Hydroxystilbamidine	11.5 ± 1.1	5.4 ± 0.5
Fostamatinib	21.0 ± 1.3	11.7 ± 0.7
Esomeprazole	13.6 ± 1.1	3.7 ± 0.4

**FIGURE 2:**
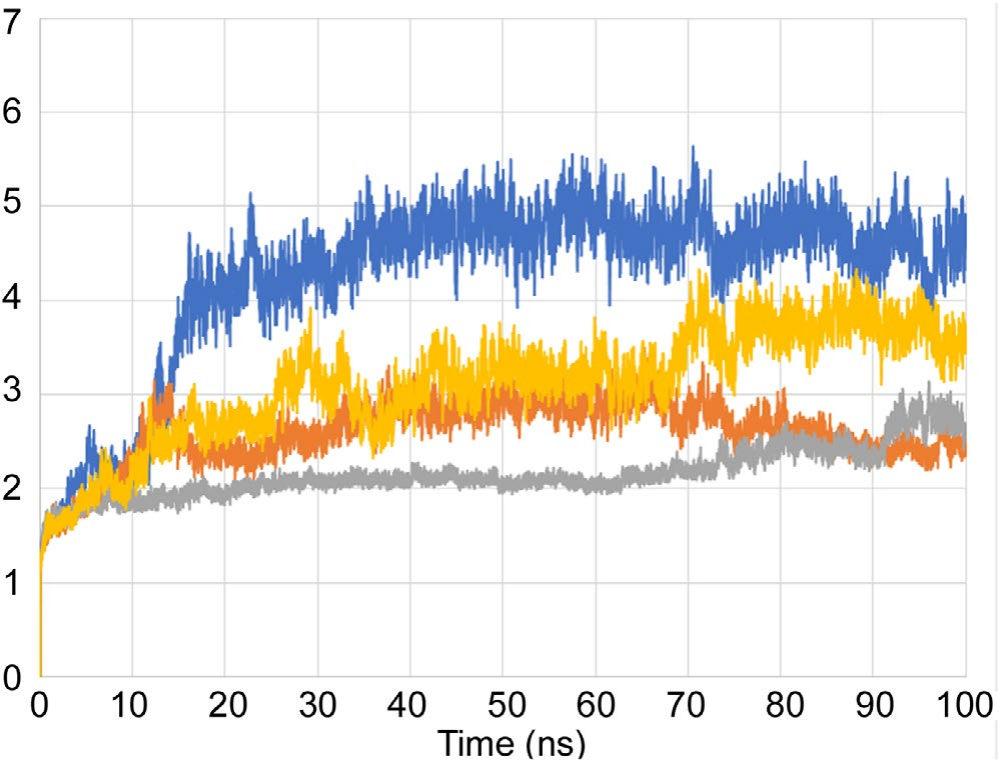
RMSD as a function of time for Mab‐SaS trajectory evaluated by Desmond. The blue curve represents the apo form, while the systems in complex with hydroxystilbamidine, fostamatinib, and esomeprazole are shown in orange, gray, and yellow, respectively.

**FIGURE 3:**
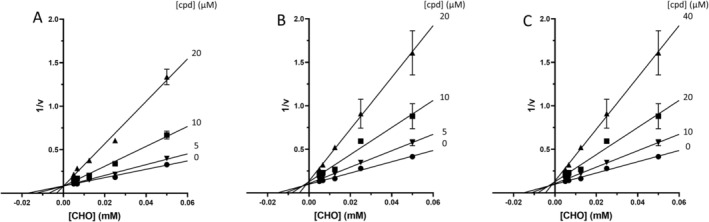
Inhibition assays of *Mab*‐SaS enzymatic activity by the three best candidates. Panel A, B, and C display the reciprocal plots of the steady‐state kinetic analyses of *Mab*‐SAS towards CHO in the presence of different concentrations of esomeprazole, hydroxystilbamidine, and fostamatinib, respectively, demonstrating the competitive behavior of the inhibitors.

**FIGURE 4:**
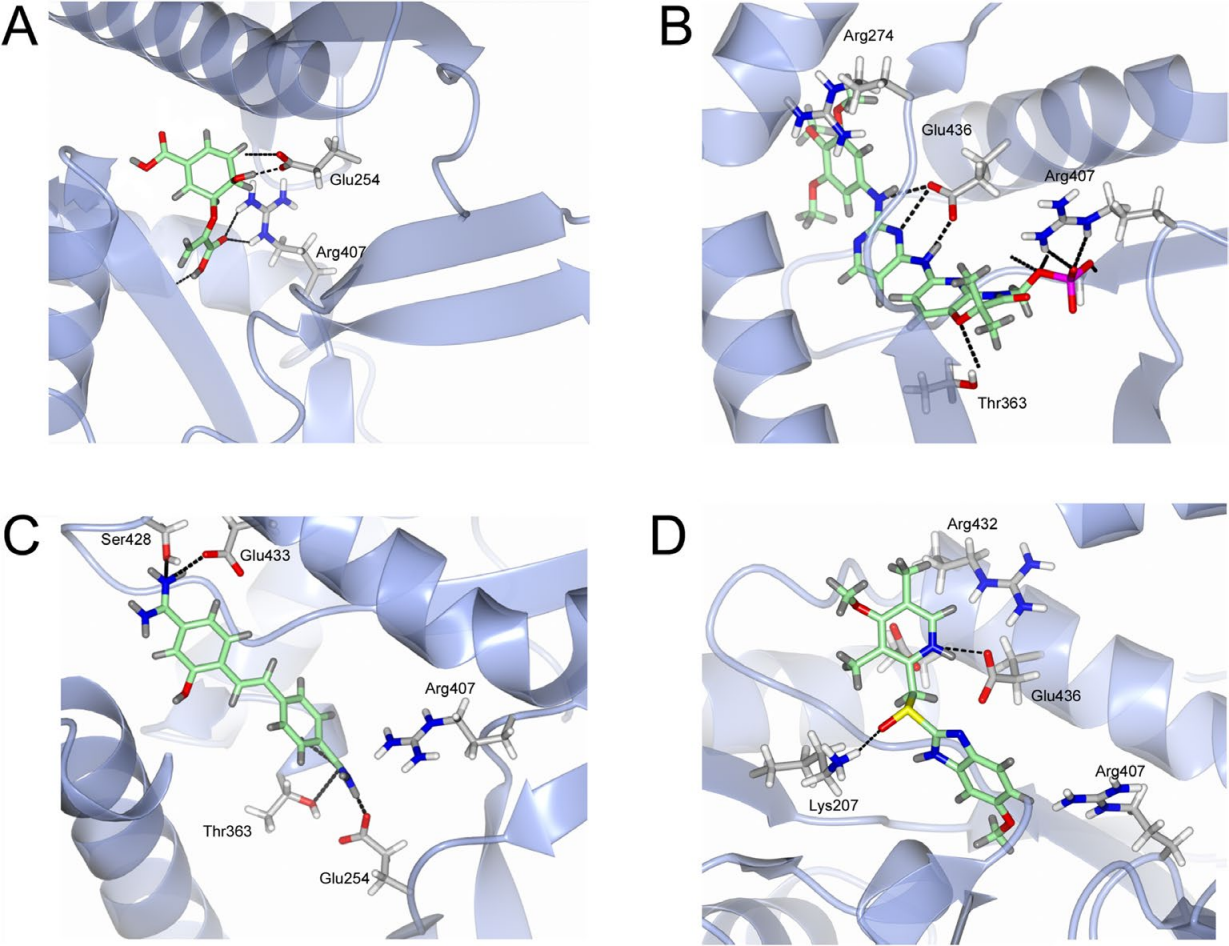
Docking scheme of (A) CHO, (B) fostamatinib, (C) hydroxystibamidine and (D) esomeprazole. CHO reacts with the catalytic Glu254, in agreement with the theoretical model, while Arg407, Thr273, and Gly272 are important for the interaction between the enzyme and the substrate. The three inhibitors interact with the same amino acids that are involved in SaS catalysis.

This data is in line with the MD analysis of the three inhibitors performed against the enzyme. According to the prediction, the drugs accommodate in the active site of Mab‐SaS, stabilizing the whole protein structure. As shown in Figure 2, the RMSD of the apo form of Mab‐SaS is higher than in complex with the three ligands, suggesting reduced flexibility upon drug binding. To further confirm that the three compounds bind to the enzyme active site, a steady‐state kinetic analysis of the enzyme in the presence of different concentrations of the inhibitors was performed. All three molecules were demonstrated to behave as competitive inhibitors with K_i_ values in the low μM range (Figure 3). This result aligns with expectations and supports the models generated from the docking analysis and molecular dynamic simulation. As illustrated in Figure 4, the compounds are positioned within the active site, where CHO interacts with the catalytic amino acids. This interaction pattern was also evidenced in previous studies on the homologous enzyme MbtI from *Mtb* and further validated in *Mab* through structural superimposition with MbtI crystal structures (Mori et al. 2020); (Mori et al. 2024). The model suggests that all three inhibitors bind to the same catalytic amino acids, but each interacts with these residues in a distinct manner, as shown in Figure 4. The phosphate group of fostamatinib establishes an ionic bond with the side chain of Arg407. A similar interaction has previously been observed between this arginine and the carboxylic group of furan‐based inhibitors (Mori et al. 2024). Additionally, other interactions with non‐catalytic amino acids, as reported in the docking scheme, likely contribute to stabilizing the inhibitor in the active site.

Hydroxystibamidine specifically interacts with the catalytic residue Glu254 through a combination of a salt bridge and a hydrogen bond.

Additionally, other strong interactions, such as two π‐cation bonds, enhance the inhibitor's stability and may prolong its residence time in the active site compared to the other two molecules. Esomeprazole forms hydrogen bonds with Gly272 and Thr273, supported by π‐interactions between the benzimidazole moiety and Lys440. This contact may contribute to impairing *Mab*‐SaS activity, potentially paving the way for a new series of SaS inhibitors upon further optimization. Finally, to assess the potential impact of these molecules on bacterial growth, their MIC on *M. smegmatis* mc^2^155 was evaluated under iron‐limiting conditions. Among the three Mab‐SaS inhibitors, only hydroxystilbamidine showed a modest effect, with a MIC of 250 μM (Figure S2).

## 3 Conclusions

Given the lengthy timeline required for developing new drugs, from the preclinical phase to the commercialization of the final product, it is crucial to shorten the drug discovery process to effectively combat the emergence of resistant strains and minimize the economic impact on society. Repurposing already approved drugs, as demonstrated in the literature, offers a viable solution (Kulkarni et al. 2023). In this study, we employed this strategy to identify novel *Mab*‐SaS inhibitors capable of disrupting the iron uptake process in *Mab* as part of an anti‐virulence approach. Among the 11 potential ligands identified through *in silico* virtual screening, three were confirmed to be highly effective competitive inhibitors of the enzyme. These results not only validate the reliability of our screening approach but also highlight the promising potential of these compounds as therapeutic agents. The identification of these strong inhibitors marks a significant advancement in our understanding of *Mab*‐SaS and underscores the feasibility of using computational methods to uncover novel drug candidates. The ability of these ligands to competitively inhibit the enzyme suggests that they can effectively disrupt the iron uptake process in *Mab*, offering a novel strategy for combating infections caused by this pathogen. Of particular interest is hydroxystilbamidine, a compound currently used in particular clinical settings to treat skin blastomycosis and known for its antifungal, antitrypanosomal, antimalarial, and carcinostatic activities (Abdeen et al. 2016); (Das and Boykin 1977); (Hermans and Keys 1983). This compound demonstrated a *K*_i_ of approximately 5 μM and exhibited modest activity against mycobacterial growth under iron‐limiting conditions. These promising results highlight the potential of this approach and lay the foundation for further experimental validation and optimization, enhancing the likelihood of developing effective treatments for mycobacterial diseases.

## 4 Experimentals

### 4.1 In Silico Studies

Maestro, a module of the Schrödinger 2025‐1 suite, was employed to construct a full‐length model of *Mab*‐SaS by including the amino acids not solved in the crystal structure, through the Protein Preparation Wizard tool and optimizing the available crystal structure (PDB ID: 8QIJ) as a template (Mori et al. 2024). Subsequently, the model was optimized through the Protein Preparation Wizard tool integrated in Maestro with standard parameters, using the OPLS_2005 force field (Reynolds et al. 2008). Water molecules and all the unnecessary atoms were eliminated during this process.

FDA‐approved drugs were retrieved by Enamine, DrugBank, and PubChem data banks (Friesner et al. 2006); (Kim et al. 2023); (Knox et al. 2024). The structures were prepared using LigPrep (Maestro). This tool generated ~3.400 conformers, considering various protonation states, stereoisomers, and possible tautomers, depending on the chemical structure of the ligands.

CHO, the natural substrate of salicylate synthases, and the ligands were docked in the *Mab*‐SaS model using the ligand‐flexible method integrated in the Glide module of Maestro (Friesner et al. 2006). For this analysis, the OPLS_2005 force field and a cubic grid with sides of 22 Å were used. The grid was intentionally generated around the whole active site to fully cover the cavity, reducing any possible missing information from this analysis.

The results were filtered based on the docking position, the docking score, and the free binding energy, estimated by Glide. Duplicates were eliminated, and the remaining compounds underwent a second docking analysis, applying the extra‐precision method to evaluate the best poses and possible steric hindrance in the cavity. At the end of the process, 14 molecules were selected for MM‐GBSA analysis.

The binding energy of the selected compounds was evaluated by the MM‐GBSA routine integrated in the Prime module of Maestro at a distance of 10 Å in the inhibitor surrounding, according to the following formula:

$$\mathrm{\Delta G}\left(\text{bind}\right)=\mathrm{\Delta G}\left(\text{solv}\right)+\mathrm{\Delta E}\left(\mathrm{MM}\right)+\mathrm{\Delta G}\left(\mathrm{SA}\right)$$

where *ΔG(bind)* corresponds to the free‐binding energy of a ligand to its receptor, *ΔG(solv)* is the change of energy of solvation, *ΔE(MM)* is the global change in minimized energy, and *ΔG(SA)* is the change in the surface area energy. In this case, *ΔG(solv)* was equal to 0 since docking was performed in void.

The Molecular Dynamics (MD) simulation of the most promising compounds was performed in Desmond, a tool integrated in Maestro. For this study, the system was built in orthorhombic shape, distant 10 Å from the Mab‐SaS model with predefined SPC solvent. K+ and PO4‐ ions have been added to the concentration of 0.15 M to simulate the in vitro conditions. The minimization was extended for 100 ps, while the full simulation was for 200 ns at 300 K and 1 bar, in isothermal‐isobaric (NPT) ensemble upon system relaxation, using the OPLS_2005 force field. RMSD of all MD has been evaluated with the trajectory analysis tool integrated in Desmond.

The root mean square deviation (RMSD) of the fluctuation of Mab‐SaS backbone during the MD has been evaluated in the absence and presence of the selected drugs according to the Desmond integrated tool.

### 4.2 Biological Assays

The recombinant *Mab*‐SaS was expressed in *E. coli* and purified as previously reported (Mori et al. 2023). Enzyme activity was determined by a direct fluorometric assay (Mori et al. 2020). Briefly, the assays were performed in 400 μL of 50 mM potassium phosphate pH 8.0, containing 5 mM MgCl_2_, and 1–2 μM *Mab*‐SaS. The reaction was started by the addition of CHO and monitored using a PerkinElmer LS3 fluorimeter (Ex. λ = 305 nm, Em. λ = 410 nm; PerkinElmer, Waltham, MA, USA).

The compounds identified by the *in silico* investigations were purchased from CymitQuimica S.L. and dissolved at a concentration of 20 mM in dimethylsulfoxide (DMSO) or water, according to their solubility. Specifically, gentamycin, cytidine, amifostine, and hydroxystilbamidine were dissolved in water, while esomeprazole, fostamatinib, S‐adenosyl‐L‐homocysteine, norepinephrine, labetalol, levolansoprazole, and nebivolol were dissolved in DMSO.

The inhibitory activity of the candidates was initially evaluated at a final concentration of 100 μM, using a subsaturating concentration of the substrate (CHO) of 70 μM. For compounds that significantly inhibited the enzyme, IC_50_ and K_i_ values were also determined. IC_50_ was calculated by measuring enzymatic activity in the presence of different compound concentrations, using (Equation 1), where A[I] is the enzyme activity at inhibitor concentration [I] and A[0] is the enzyme activity without inhibitor.

(1) $$A_{\left[I\right]}=A_{\left[0\right]}\times \left(1-\frac{\left[I\right]}{\left[I\right]+{\mathrm{IC}}_{50}}\right)$$

The inhibition constant values (*K*_i_) were determined by assaying *Mab*‐SaS at different substrate and compound concentrations and using an adapted equation for competitive inhibition (Equation 2) with GraphPad 8.0 software.

(2) $$v=\frac{V_{\max}\left[S\right]}{\left[S\right]+K_{m}\left(1+\frac{\left[I\right]}{K_{i}}\right)}$$

To verify that the compounds are not PAINs, their ability to inhibit enzymatic activity was assayed in the presence of 0.1 mg/mL bovine serum albumin (BSA) or in the presence of 0.01% (v/v) Triton X‐100 to confirm that they did not promote protein aggregation, and in the presence of 100 mM of 1,4‐dithio‐DL‐threitol (DTT) to exclude reaction with cysteines (Mori et al. 2020).

Antimycobacterial activity of the best compounds was evaluated by REMA assay in iron‐limiting chelated Sauton's medium on *M. smegmatis* MC^2^ 155 (Siegrist et al. 2009; Mehra and Philips 2014). This microorganism is widely used as a non‐pathogenic model for *Mab*, since it shares similar biological characteristics and replicates more rapidly than other mycobacteria. By using this non‐pathogenic microorganism, we could efficiently study processes related to Mab, while minimizing the hazards and technical difficulties of working directly with a pathogenic strain.

## Conflicts of Interest

The authors declare no conflicts of interest.

## Supporting information


**Figure S1.** Interaction schemes of the docking of chorismic acid and the 3 approved drugs proved as inhibitors of Mab‐SaS: Hydroxystilbamidine, Fostamatinib, and Esomeprazole.
**Table S1.** In vitro inhibitory effects of the compounds obtained by docking analyses against *Mab*‐SaS enzyme.
**Figure S2.** MIC determination of hydroxystilbamidine, fostamatinib, and esomeprazole against *M. smegmatis* MC^2^ 155 growth.

## Data Availability

The data that supports the findings of this study are available from the corresponding author upon request.
