# Supplementary material for: Repurposing of FDA‐Approved Drugs to Disrupt Iron Uptake in Mycobacterium abscessus: Targeting Salicylate Synthase as a Novel Approach
Source: Chem Biol Drug Des. 2025 Jul 29;106(2):e70162. doi: 10.1111/cbdd.70162 (PMC12305296; doi:10.1111/cbdd.70162)

**Figure S1**: Interaction schemes of the docking of chorismic acid and the 3 approved drugs proved as inhibitors of Mab-SaS: Hydroxystilbamidine, Fostamatinib, and Esomeprazole.

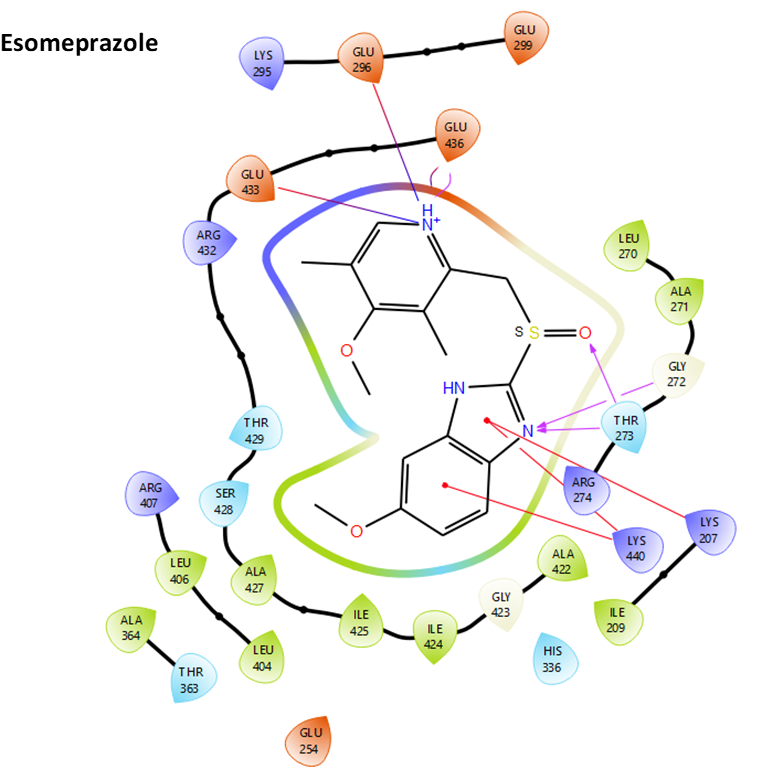


| Suppl. Table 1. In vitro inhibitory effects of the compounds obtained by docking analyses against *Mab*-SaS enzyme. | |
| --- | --- |
| Compound | residual activity at 100 μM  (%) |
| Nebivolol | 82.3 ± 1.3 |
| Hydroxystilbamidine | 0.5 ± 0.3 |
| Cytidine | 88.8 ± 2.5 |
| Gentamycin | 98.8 ± 5.7 |
| Norepinephrine | 75.5 ± 1.4 |
| Fostamatinib | 11.6 ± 0.7 |
| Labetalol | 47.0 ± 2.1 |
| Levolansoprazole | 62.8 ± 1.7 |
| Esomeprazole | 3.3 ± 1.7 |
| Amifostine | 78.1 ± 3.2 |
| S-adenosyl-L-homocysteine | 101.8 ± 3.8 |

Results are Mean ± SD of three different experiments

**Figure S2**: MIC determination of hydroxystilbamidine, fostamatinib, and esomeprazole against *M. smegmatis* MC^2^ 155 growth.


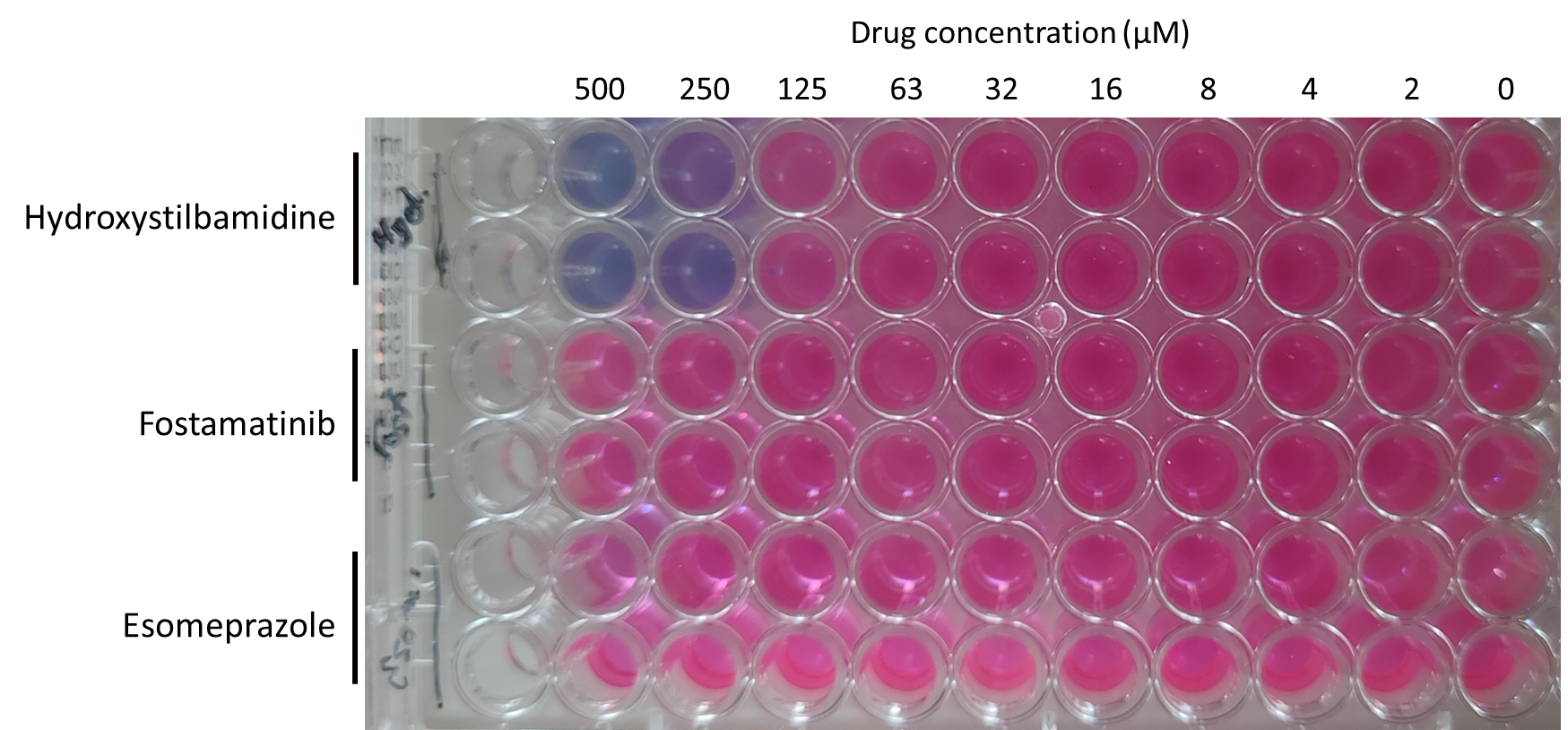

Supplement: Supplementary file 1 — Figure S1. Interaction schemes of the docking of chorismic acid and the 3 approved drugs proved as inhibitors of Mab‐SaS: Hydroxystilbamidine, Fostamatinib, and Esomeprazole. Table S1. In vitro inhibitory effects of the compounds obtained by docking analyses against Mab‐SaS enzyme. Figure S2. MIC determination of hydroxystilbamidine, fostamatinib, and esomeprazole against M. smegmatis MC2 155 growth. [file CBDD-106-e70162-s001.docx]
